# Self-reported eating habits and dyslipidemia in men aged 20–39 years: the Japan Environment and Children’s Study

**DOI:** 10.1265/ehpm.23-00008

**Published:** 2023-07-05

**Authors:** Meishan Cui, Satoyo Ikehara, Kimiko Ueda, Kazumasa Yamagishi, Hiroyasu Iso, the Japan Environment and Children’s Study Group

**Affiliations:** 1 Public Health, Department of Social Medicine, Osaka University Graduate School of Medicine, Osaka, Japan; 2 Osaka Maternal and Child Health Information Center, Osaka Women’s and Children’s Hospital, Osaka, Japan; 3 Department of Public Health Medicine, Faculty of Medicine, and Health Services Research and Development Center, University of Tsukuba, Tsukuba, Japan; 4 Institute for Global Health Policy Research, Bureau of International Health Cooperation, National Center for Global Health and Medicine, Tokyo, Japan

**Keywords:** Dyslipidemia, Elevated TC, Elevated TG, Lipoprotein cholesterol, Self-reported eating habit, Young adult men

## Abstract

**Background and aims:**

Unhealthy eating behaviors, including eating fast, eating after satiety, skipping breakfast, and eating out are common among men aged 20–39 years. In this cross-sectional study, we aimed to examine the association between self-reported eating habits and the prevalence of dyslipidemia.

**Methods:**

The participants of this study were 38,233 men aged 20–39 years, whose food consumption frequency related information was collected through a questionnaire. Dyslipidemia was defined as total cholesterol (TC) ≥190 mg/dL, fasting triglyceride (TG) ≥150 mg/dL and non-fasting TG ≥175 mg/dL, high-density lipoprotein cholesterol (HDL-C) <40 mg/dL, low-density lipoprotein cholesterol (LDL-C) ≥140 mg/dL. Odds ratios (ORs) and 95% confidence intervals were calculated relative to healthy eating habits using logistic regression, after adjustment for age, study unit, and other potential confounding factors.

**Results:**

Moderate and fast speeds were associated with a higher prevalence of reduced HDL-C (by 27% and 26%, respectively) compared to slow speeds. Eating after satiety was associated with a higher prevalence of elevated TC (by 16%) and elevated TG (by 11%), elevated LDL-C (by 21%). Breakfast eating of 1–4 times/week and <1 time/week were associated with a higher prevalence of elevated TC (by 11% and 16%, respectively) and elevated LDL-C (by 21% and 38%, respectively) compared to that of ≥5 times/week. Eating out of ≥5 times/week was associated with a 13% higher prevalence of elevated TG.

**Conclusions:**

All of four unhealthy eating habits were associated with a higher prevalence of dyslipidemia in men aged 20–39 years.

## Introduction

Cardiovascular disease (CVD) is among the leading causes of death and is becoming more prevalent worldwide [1]. Active and comprehensive management of cardiovascular risk factors is crucial to reduce the incidence and mortality of CVD. Dyslipidemia is a critical risk factor for atherosclerosis [2–5], while the burden of dyslipidemia has increased in prevalence among young adults owing to the ubiquity of unhealthy lifestyles, including poor eating habits [2, 6]. Moreover, dyslipidemia in young adults has been associated with the incidence of cardiovascular events in later life [2, 7] while it presents with no apparent symptoms, which may complicate or delay the diagnosis, especially for young adults. In addition, eating a nutritionally balanced diet suggested to be effective in controlling for cardiovascular risk factors, such as obesity, dyslipidemia, and hypertension, among male workers aged 20 to 59 years [8]. Therefore, identifying modifiable risk factors, such as dietary habits, for dyslipidemia is crucial as it would help prevent and reduce the risk of dyslipidemia in early adulthood.

Nutritional imbalances, such as excessive intake of saturated fatty acids and an insufficient intake of micronutrients and vitamins, coupled with lifestyle habits such as smoking, alcohol, and physical inactivity, have been recognized as important modifiable risk factors for dyslipidemia [2]. Eating speed and satiation were associated with the prevalence of being overweight [9] and having diabetes [10]. However, few studies have examined the associations of unhealthy eating habits, such as eating fast, eating after satiety, skipping breakfast, and eating out with dyslipidemia. Furthermore, to our knowledge, no study has focused on the association between eating habits and dyslipidemia in young adults. In this study, we tested the hypothesis that eating fast, eating after satiety, skipping breakfast, and eating out are associated with a higher prevalence of dyslipidemia among a large sample of men aged 20–39 years, under the Japan Environment and Children’s Study (JECS).

## Material and methods

### Study population

**Fig. 1:**
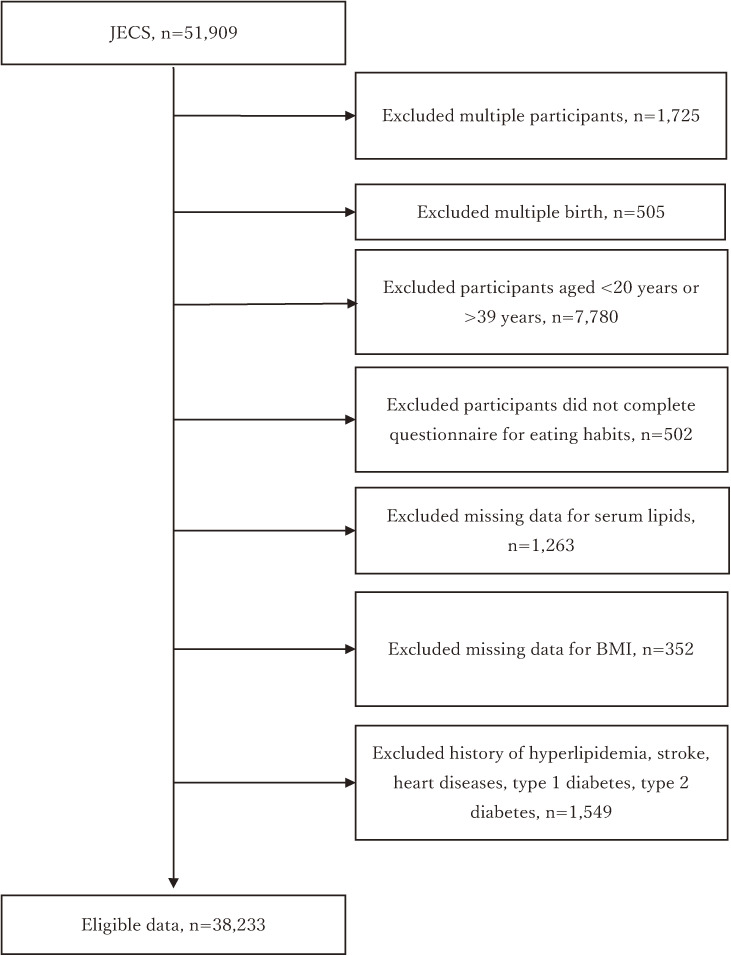
Flowchart of the process of selecting participants for analysis.

This was a nationwide, government-funded, prospective birth cohort study. The JECS protocol has been described in detail elsewhere [11, 12]. The study covers 97,454 pregnant females and 49,679 male partners between January 2011 and March 2014. Our study subjects were restricted to men because women in the JECS were at pregnancy so that blood lipid profiles were affected by pregnancy perse. After excluding men without blood sample and body mass index (BMI) data, those who did not complete the questionnaire covering eating habits, and those with a history of hyperlipidemia, stroke, heart disease, type-1 diabetes, or type-2 diabetes, a total of 38,233 men aged 20–39 years were eligible for this analysis (Fig. 1).

The JECS protocol was reviewed and approved by the Ministry of the Environment’s Institutional Review Board on epidemiological studies and the ethics committees of all participating institutions. All participants provided written informed consent.

### Data collection

The self-reported questionnaire on age, weight, height, occupation, smoking status, alcohol intake, dietary intake, and self-reported eating habits and non-fasting blood samples were collected for men between his partner’s early pregnancy. The information on education was obtained from his partner’s questionnaires at second/third trimester. Blood samples were stored at −80 °C freezers until chemical analysis. Serum total cholesterol (TC), low-density lipoprotein cholesterol (LDL-C), high-density lipoprotein cholesterol (HDL-C) and triglycerides (TG) were analyzed enzymatically using a 7700 clinical chemistry/immunoassay hybrid analyzer (Hitachi High-Technologies Co., Ltd, Tokyo, Japan).

### Exposure and outcome measures

We used the food frequency questionnaire on diet history to assess participants’ eating habits during the previous year [13, 14]. The participants were asked about their eating speeds (very slow, slow, medium, fast, and very fast), whether they usually eat after satiety (yes or no), their breakfast intake frequency (<1 time/month, 1–3 times/month, 1–2 times/week, 3–4 times/week, 5–6 times/week, every day), and their frequency of eating out (<1 time/month, 1–3 times/month, 1–2 times/week, 3–4 times/week, 5–6 times/week, every day). We re-categorized eating speed as slow (combined very slow and slow), moderate, and fast (combined very fast and fast); frequency of breakfast intake as ≥5 times/week, 1–4 times/week, and <1 time/week; and frequency of eating out as <1 time/week, 1–4 times/week, and ≥5 times/week.

Fasting was not required before blood sampling (fasting hour <10 hours: 86.6%, fasting hour ≥10 hours: 13.4%). We used cut-off points from the Japan Atherosclerosis Society Guidelines for Prevention of Atherosclerotic Cardiovascular Diseases [15]; Elevated TC was defined as TC ≥190 mg/dL, elevated TG as TG ≥150 mg/dL for fasting and ≥175 mg/dL for non-fasting, elevated LDL-C as LDL-C ≥140 mg/dL, and reduced HDL-C as HDL-C <40 mg/dL.

### Statistical analysis

This study was based on the dataset: jecs-ag-20160424. Missing data for risk factor variables were deleted from the analyses. We present the characteristics of the study participants according to self-reported eating habits as means or proportions. To examine differences in the mean values of lipid profiles and the confounding factors according to eating habits, we used the analysis of covariance and used chi-square tests to assess the proportions of potential confounding factors. Logistic regression analysis was used to calculate odds ratios (ORs) and 95% confidence intervals (CIs) of dyslipidemia according to eating habits, by creating 4 separate models: Model 1 included age and study unit (20 areas). Model 2 further included BMI (continuous). Model 3 further included model 2 and education (less than university, university or higher), sedentary workers (yes or no), smoking status (current smoker or not), alcohol intake (continuous), total energy (continuous), fasting (yes or no), dietary intakes of saturated fat (continuous), cholesterol (continuous), and n-3 polyunsaturated fatty acids (continuous), as well as eating speed, eating full, skipping breakfast, and eating out mutually. Sedentary workers included work profiles with less physical activity, including managers, professionals and technicians, clerical support work, driving, and machine operations, full-time homemakers, students, and unemployed workers. The analysis of trend was performed by allocating 0 to 4 for none to four unhealthy eating habits, respectively. All statistical analyses were performed using SAS version 9.4 (SAS Institute Inc. Cary, NC, USA). All probability values for statistical tests were two-tailed, and statistical significance was set at *p* < 0.05.

## Results

As shown in Table 1, the mean levels of TC, TG, HDL-C and LDL-C were 190.8 mg/dL, 155.9 mg/dL, 56.4 mg/dL, and 114.5 mg/dL, respectively. The prevalence of elevated TC, elevated TG, reduced HDL-C, and elevated LDL-C were 48.5%, 30.2%, 6.5%, and 18.2%, respectively (data not shown). Compared with slow eaters, fast eaters were older, less educated, included fewer sedentary workers and more smokers, had a higher prevalence of being overweight and a greater intake of alcohol and calories. Compared with young men who did not eat after satiety, those who ate after satiety were less educated, included fewer sedentary workers, had a higher prevalence of being overweight, and had a greater calorie intake. Compared with men who ate breakfast ≥5 times/week, men who skipped breakfast and ate breakfast <1 time/week were younger, less educated, less likely to be sedentary workers, more likely to be smokers, less overweight, consumed more alcohol but fewer calories. Compared with people who ate out <1 time/week, those who ate out ≥5 times/week were older, more educated, more likely to be sedentary workers, less likely to smoke, more likely to be overweight, and consumed more alcohol and calories.

**Table 1:** Age-adjusted characteristics for men aged 20–39 years according to eating habits.

	**Total**	**Eating speed**			**Eating after satiety**		**Eating breakfast**			**Eating out**		
			
		**Slow**	**Moderate**	**Fast**	**No**	**Yes**	**≥5 times/wk**	**1–4 times/wk**	**<1 time/wk**	**<1 time/wk**	**1–4 times/wk**	**≥5 times/wk**
No. of participants	38,233	3,278	9,186	25,769	10,987	27,246	24,138	7,916	6,179	16,584	16,328	5,321
Age, mean (SE)	31.3 (0.02)	31.2 (0.1)	31.4 (0.05)	31.3 (0.03)	31.3 (0.04)	31.3 (0.03)	31.7 (0.03)	30.8 (0.05)	30.4 (0.1)	31.1 (0.03)	31.2 (0.03)	32.3 (0.06)
University or higher, %	33.8	40.6	32.4	33.5	35.4	33.2	37.2	30.4	25.2	27.1	37.1	45.0
Sedentary workers, %	48.7	53.5	48.4	48.2	49.6	48.3	51.3	44.4	43.9	45.9	50.8	50.8
Current smokers, %	43.3	34.1	42.8	44.7	43.8	43.2	35.6	51.3	63.6	46.7	40.2	42.7
Alcohol intake, g/week (SE)	95.8 (1.0)	81.3 (3.3)	95.9 (2.0)	97.7 (1.2)	93.8 (1.8)	96.7 (1.2)	88.0 (1.2)	103.3 (2.1)	116.9 (2.4)	94.7 (1.5)	94.6 (1.5)	103.1 (2.6)
Mean BMI, kg/m^2^ (SE)	23.3 (0.02)	21.9 (0.06)	22.5 (0.04)	23.8 (0.02)	21.4 (0.03)	24.1 (0.02)	23.4 (0.02)	23.4 (0.04)	23.0 (0.04)	23.3 (0.03)	23.4 (0.03)	23.4 (0.05)
Overweight (BMI ≥25), %	25.9	12.1	17.0	30.8	7.0	33.5	26.2	26.9	23.5	25.2	26.4	26.5
Mean TC, mg/dL (SE)	190.8 (0.2)	187.0 (0.6)	189.2 (0.3)	191.8 (0.2)	184.7 (0.3)	193.2 (0.2)	189.7 (0.2)	192.4 (0.4)	192.6 (0.4)	190.1 (0.3)	191.4 (0.3)	190.7 (0.4)
Mean TG, mg/dL (SE)	155.9 (0.6)	134.9 (2.1)	146.0 (1.2)	162.1 (0.7)	131.7 (1.1)	165.6 (0.7)	155.8 (0.8)	159.8 (1.3)	151.5 (1.5)	153.6 (0.9)	156.8 (0.9)	160.2 (1.6)
Mean HDL-C, mg/dL (SE)	56.4 (0.06)	59.1 (0.2)	57.5 (0.1)	55.7 (0.1)	58.8 (0.1)	55.4 (0.1)	56.6 (0.1)	55.9 (0.1)	56.1 (0.2)	56.6 (0.1)	56.4 (0.1)	55.8 (0.2)
Mean LDL-C, mg/dL (SE)	114.5 (0.1)	110.2 (0.5)	112.8 (0.3)	115.6 (0.2)	108.5 (0.3)	116.9 (0.2)	113.2 (0.2)	116.4 (0.3)	117.1 (0.4)	113.9 (0.2)	115.1 (0.2)	114.7 (0.4)
Fasting hour, hours (SE)	4.3 (0.2)	4.3 (0.07)	4.3 (0.04)	4.3 (0.02)	4.3 (0.04)	4.3 (0.02)	3.8 (0.02)	4.7 (0.04)	5.8 (0.05)	4.3 (0.03)	4.3 (0.03)	4.3 (0.05)
Carbohydrates intake, %energy (SE)	56.0 (0.05)	56.2 (0.16)	56.1 (0.10)	55.9 (0.06)	56.0 (0.09)	56.0 (0.06)	56.1 (0.06)	56.0 (0.11)	56.0 (0.12)	56.4 (0.07)	55.6 (0.07)	55.9 (0.13)
Protein intake, %energy (SE)	12.5 (0.01)	12.6 (0.04)	12.4 (0.02)	12.5 (0.01)	12.5 (0.02)	12.5 (0.01)	12.6 (0.01)	12.3 (0.03)	12.0 (0.03)	12.4 (0.02)	12.6 (0.02)	12.5 (0.03)
Fat intake, %energy (SE)	25.5 (0.03)	25.8 (0.13)	25.3 (0.08)	25.5 (0.05)	25.4 (0.07)	25.5 (0.05)	25.7 (0.05)	25.2 (0.08)	24.8 (0.10)	25.1 (0.06)	25.9 (0.06)	25.4 (0.10)
Saturated fatty acid intake, g/day (SE)	21.1 (0.1)	20.8 (0.3)	20.2 (1.7)	21.5 (0.1)	19.8 (0.2)	21.6 (0.1)	21.7 (0.1)	20.4 (0.2)	19.5 (0.2)	20.4 (0.1)	21.7 (0.1)	21.3 (0.2)
Monounsaturated fatty acid intake, g/day (SE)	25.7 (0.1)	24.8 (0.3)	24.4 (0.2)	26.3 (0.1)	24.0 (0.2)	26.4 (0.1)	26.1 (0.1)	25.4 (0.2)	24.7 (0.2)	24.8 (0.1)	26.5 (0.1)	26.1 (0.3)
Polyunsaturated fatty acids intake, g/day (SE)	13.4 (0.1)	13.0 (0.2)	12.8 (0.1)	13.7 (0.1)	12.5 (0.1)	13.7 (0.1)	13.7 (0.1)	13.9 (0.1)	12.6 (0.1)	13.1 (0.1)	13.7 (0.1)	13.4 (0.1)
n-3 polyunsaturated fatty acid intake, g/day (SE)	2.1 (0.01)	2.0 (0.03)	2.0 (0.02)	2.1 (0.01)	2.0 (0.02)	2.2 (0.01)	2.1 (0.01)	2.1 (0.02)	2.0 (0.02)	2.1 (0.01)	2.2 (0.01)	2.1 (0.02)
n-6 polyunsaturated fatty acid intake, g/day (SE)	11.3 (0.04)	10.9 (0.1)	10.7 (0.1)	11.5 (0.04)	10.5 (0.1)	11.5 (0.04)	11.5 (0.04)	11.0 (0.1)	10.6 (0.1)	11.0 (0.1)	11.5 (0.1)	11.3 (0.1)
Cholesterol intake, mg/day (SE)	323.7 (1.7)	316.4 (5.7)	305.9 (3.4)	330.9 (2.0)	298.6 (3.1)	333.8 (2.0)	336.3 (0.1)	307.3 (3.6)	295.3 (4.1)	321.6 (2.5)	330.5 (2.5)	309.1 (4.5)
Energy intake, kcal/day (SE)	2312 (6)	2240 (19)	2221 (11)	2353 (7)	2174 (10)	2368 (7)	2343 (7)	2291 (12)	2217 (14)	2267 (8)	2344 (9)	2352 (15)

SE, standard error.

Multivariable adjusted ORs and 95% CIs of dyslipidemia according to eating habits are shown in Table 2. Both moderate and fast eaters showed higher age- and study unit-adjusted ORs for all types of dyslipidemia (Model 1). When adjusting further for BMI (Model 2), the positive association remained statistically significant for elevated LDL-C and reduced HDL-C, but not for elevated TC and elevated TG. After further adjustment for other cardiovascular risk factors and eating habits mutually (Model 4), the association between eating fast and reduced HDL-C remained statistically significant. Eating after satiety was associated with elevated TC, elevated TG, and elevated LDL-C in all models. The frequency of eating breakfast was inversely associated with elevated TC and elevated LDL-C in all models. The frequency of eating out was positively associated with elevated TG in all models.

**Table 2:** Odds ratios and 95% confidence intervals of dyslipidemia according to eating habits.

	**Eating speed**			**Eating after satiety**		**Eating breakfast**			**Eating out**		
			
	**Slow**	**Moderate**	**Fast**	**No**	**Yes**	**≥5 times/wk**	**1–4 times/wk**	**<1 time/wk**	**<1 time/wk**	**1–4 times/wk**	**≥5 times/wk**
No. of participants	3,278	9,186	25,769	10,987	27,246	24,138	7,916	6,179	16,584	16,328	5,321
Elevated TC											
No. of cases (%)	1432 (43.7)	4303 (46.8)	12812 (49.7)	4469 (40.7)	14078 (51.7)	11657 (48.3)	3903 (49.3)	2987 (48.3)	7851 (47.3)	8025 (49.2)	2671 (50.2)
Model 1^a^	1.00	1.11 (1.02–1.20)	1.26 (1.17–1.36)	1.00	1.58 (1.51–1.66)	1.00	1.13 (1.07–1.19)	1.11 (1.05–1.18)	1.00	1.07 (1.03–1.12)	1.04 (0.97–1.10)
Model 2^b^	1.00	1.04 (0.95–1.13)	1.01 (0.93–1.09)	1.00	1.17 (1.12–1.23)	1.00	1.13 (1.07–1.19)	1.17 (1.11–1.25)	1.00	1.06 (1.01–1.11)	1.01 (0.95–1.08)
Model 3^c^	1.00	1.04 (0.95–1.13)	0.97 (0.89–1.05)	1.00	1.16 (1.10–1.22)	1.00	1.11 (1.05–1.17)	1.16 (1.09–1.24)	1.00	1.05 (0.99–1.10)	1.01 (0.95–1.08)
Elevated TG											
No. of cases (%)	756 (23.1)	2416 (26.3)	8358 (32.4)	2328 (21.2)	9202 (33.8)	7360 (30.5)	2417 (30.5)	1753 (28.4)	4842 (29.2)	4944 (30.3)	1744 (32.8)
Model 1^a^	1.00	1.17 (1.06–1.29)	1.55 (1.42–1.70)	1.00	1.84 (1.74–1.94)	1.00	0.96 (0.90–1.01)	0.73 (0.68–0.78)	1.00	1.03 (0.98–1.09)	1.14 (1.06–1.22)
Model 2^b^	1.00	1.03 (0.94–1.14)	1.10 (1.00–1.20)	1.00	1.13 (1.07–1.20)	1.00	1.05 (0.99–1.11)	1.03 (0.97–1.10)	1.00	1.03 (0.98–1.09)	1.10 (1.03–1.18)
Model 3^c^	1.00	0.99 (0.89–1.09)	1.02 (0.93–1.12)	1.00	1.11 (1.05–1.18)	1.00	1.03 (0.97–1.10)	1.01 (0.94–1.09)	1.00	1.06 (1.01–1.12)	1.13 (1.05–1.22)
Elevated LDL-C											
No. of cases (%)	446 (13.6)	1528 (16.6)	4966 (19.3)	1382 (12.6)	5558 (20.4)	4121 (17.1)	1551 (19.6)	1268 (20.5)	2901 (17.5)	3053 (18.7)	986 (18.5)
Model 1^a^	1.00	1.24 (1.11–1.39)	1.50 (1.35–1.67)	1.00	1.80 (1.69–1.92)	1.00	1.26 (1.18–1.35)	1.37 (1.27–1.47)	1.00	1.08 (1.02–1.14)	1.01 (0.93–1.09)
Model 2^b^	1.00	1.15 (1.02–1.29)	1.14 (1.03–1.27)	1.00	1.24 (1.16–1.33)	1.00	1.27 (1.18–1.35)	1.46 (1.36–1.57)	1.00	1.06 (1.00–1.12)	0.97 (0.89–1.05)
Model 3^c^	1.00	1.13 (0.99–1.27)	1.07 (0.95–1.19)	1.00	1.21 (1.13–1.31)	1.00	1.21 (1.12–1.30)	1.38 (1.27–1.49)	1.00	1.07 (1.01–1.14)	0.97 (0.89–1.06)
Reduced HDL-C											
No. of cases (%)	121 (3.7)	506 (5.5)	1841 (7.1)	448 (4.1)	2020 (7.4)	1520 (6.3)	542 (6.9)	406 (6.6)	1086 (6.6)	999 (6.1)	383 (7.2)
Model 1^a^	1.00	1.48 (1.21–1.82)	1.98 (1.64–2.39)	1.00	1.88 (1.70–2.09)	1.00	1.11 (1.00–1.23)	1.06 (0.94–1.18)	1.00	0.94 (0.86–1.02)	1.11 (0.98–1.25)
Model 2^b^	1.00	1.36 (1.11–1.67)	1.42 (1.17–1.72)	1.00	1.18 (1.05–1.32)	1.00	1.10 (0.99–1.22)	1.12 (0.99–1.26)	1.00	0.91 (0.83–1.00)	1.07 (0.94–1.21)
Model 3^c^	1.00	1.27 (1.03–1.56)	1.26 (1.04–1.53)	1.00	1.10 (0.98–1.24)	1.00	1.08 (0.97–1.21)	1.08 (0.95–1.23)	1.00	0.94 (0.85–1.03)	1.09 (0.96–1.25)

a Model 1: adjusted for age and study unit.

b Model 2: model 1 and further adjusted for BMI.

c Model 3: model 2 and further adjusted for education, sedentary workers, smoking status, alcohol intake, BMI, fasting hours, total energy, dietary intakes of saturated fat, cholesterol, and n-3 polyunsaturated fatty acids, as well as eating speed, eating full, skipping breakfast, and eating out mutually.

We examined the association between the number of unhealthy eating habits and dyslipidemia (Table 3). Unhealthy eating habits included fast or moderate eating speeds, eating after satiety, eating breakfast ≤4 times/week, and eating out ≥5 times/week. Only 2.4% of participants had no unhealthy eating habits, and 79.7% of participants had ≥2 unhealthy eating habits. After multivariable adjustment, the number of unhealthy habits was positively associated with all types of dyslipidemia.

**Table 3:** Odds ratios and 95% confidence intervals of dyslipidemia according to number of unhealthy eating habits.

	**None**	**One**	**Two**	**Three**	**Four**	**p for trend**
No. of participants	929	6,845	18,128	10,808	1,523	
Elevated TC						
No. of cases (%)	331 (35.6)	2892 (42.3)	8932 (49.3)	5570 (51.5)	822 (54.0)	
Model 1^a^	1.00	1.31 (1.13–1.52)	1.78 (1.55–2.05)	2.02 (1.76–2.33)	2.13 (1.79–2.52)	<0.001
Model 2^b^	1.00	1.19 (1.03–1.37)	1.30 (1.13–1.50)	1.42 (1.23–1.64)	1.47 (1.23–1.75)	<0.001
Model 3^c^	1.00	1.18 (1.02–1.38)	1.29 (1.11–1.49)	1.39 (1.20–1.62)	1.45 (1.21–1.74)	<0.001
Elevated TG						
No. of cases (%)	155 (16.7)	1537 (22.5)	5709 (31.5)	3581 (33.1)	548 (36.0)	
Model 1^a^	1.00	1.42 (1.18–1.71)	2.20 (1.83–2.64)	1.19 (1.82–2.63)	2.28 (1.84–2.82)	<0.001
Model 2^b^	1.00	1.19 (0.99–1.44)	1.32 (1.10–1.58)	1.37 (1.14–1.64)	1.47 (1.19–1.81)	<0.001
Model 3^c^	1.00	1.16 (0.96–1.41)	1.27 (1.05–1.53)	1.30 (1.08–1.57)	1.45 (1.17–1.81)	<0.001
Elevated LDL-C						
No. of cases (%)	87 (9.4)	865 (12.6)	3315 (18.3)	2330 (21.6)	343 (22.5)	
Model 1^a^	1.00	1.39 (1.10–1.75)	2.18 (1.74–2.73)	2.75 (2.20–3.45)	2.80 (2.17–3.60)	<0.001
Model 2^b^	1.00	1.23 (0.97–1.55)	1.48 (1.18–1.85)	1.79 (1.43–2.25)	1.77 (1.37–2.28)	<0.001
Model 3^c^	1.00	1.22 (0.96–1.55)	1.43 (1.13–1.80)	1.68 (1.32–2.13)	1.68 (1.29–2.19)	<0.001
Reduced HDL-C						
No. of cases (%)	25 (2.7)	287 (4.2)	1205 (6.7)	803 (7.4)	148 (9.7)	
Model 1^a^	1.00	1.56 (1.03–2.37)	2.55 (1.71–3.81)	2.88 (1.93–4.32)	3.86 (2.50–5.94)	<0.001
Model 2^b^	1.00	1.33 (0.88–2.02)	1.55 (1.03–2.32)	1.67 (1.11–2.50)	2.16 (1.39–3.34)	<0.001
Model 3^c^	1.00	1.19 (0.78–1.80)	1.33 (0.88–2.00)	1.39 (0.92–2.09)	1.87 (1.20–2.92)	<0.001

a Model 1: adjusted for age and study unit.

b Model 2: model 1 and further adjusted for BMI.

c Model 3: model 2 and further adjusted for education, sedentary workers, smoking status, alcohol intake, BMI, fasting hours, total energy, and dietary intakes of saturated fat, cholesterol, n-3 polyunsaturated fatty acids.

## Discussion

In this large cross-sectional study of men aged 20–39 years, medium and fast eaters had the higher prevalence of reduced HDL-C than slow eaters. Men who ate after satiety showed the higher prevalence of elevated TC and elevated TG, and elevated LDL-C compared with those who did not. Men who skipped breakfast showed the higher prevalence of elevated TC and elevated LDL-C compared to those who ate breakfast regularly. Men who ate out regularly had the higher prevalence of elevated TG compared to those who did not. The number of unhealthy eating habits was positively associated with all types of dyslipidemia in a dose-response fashion.

Some of our results were consistent with the findings from a cross-sectional analysis of 4,819 Korean men aged 20–80 years [16] and a cohort study of 8,941 Japanese men and women aged 40–75 years [17] that the higher prevalence of reduced HDL-C was observed in fast eaters. Another cross-sectional study of 4,464 Chinese men aged 18–65 years [18] reported that eating speed was associated with the higher prevalence of elevated TG and reduced HDL-C.

Several mechanisms can explain the association between fast eating behaviors and the higher prevalence of reduced HDL-C. Eating fast has been associated with being overweight [9, 16–19]. In the present study, after adjusting for BMI, the association of eating fast and dyslipidemia were weakened, and no longer statistically significant for elevated TC and elevated TG. Eating fast and chewing less frequently reduce the production of glucagon-like peptide-1 (GLP-1) [20], which may contribute to increase HDL-C level [21].

Overfeeding showed changes in mean levels of blood lipids [22–24]. Eating after achieving satiety or overeating increases stored fat, leading to elevated TG through de novo lipogenesis [25]. Habitually overeating results in excessive calorie intake and increased weight gain, leading to increased insulin resistance [22, 24, 25], which enhances the hepatic synthesis of triglyceride-rich VLDL particles, VLDL remnants, and TG. These situations contributed to a down-regulation of hepatic Apo B/E receptor number, leading to elevated LDL-C levels [22, 25].

In this study, skipping breakfast was associated with elevated TC and elevated LDL-C, which was consistent with the finding from a recent meta-analysis of three randomized controlled trials, reporting that skipping breakfast was associated with increased LDL-C; the weighted mean difference (95% CI) was 9.24 (2.18–16.30) mg/dL, compared to not skipping breakfast [26]. In addition, skipping breakfast was associated with higher blood insulin levels [27]. Insulin stimulates 3-hydroxy-3-methyl-glutaryl coenzyme-A reductase, resulting in higher TC, as well as LDL-C concentrations [28, 29].

We have no good explanation for the association between eating out and reduced HDL-C in the present study. According to a systematic review of 27 cross-sectional studies and two prospective studies [30], eating out was positively associated with higher energy intake from dietary fat and lower dietary quality. The lower dietary quality score was associated with higher levels of TG, as well as TC and LDL-C, but not the lower levels of HDL-C [31].

Few studies have reported how the accumulation of unhealthy eating habits affect blood lipid levels. In the present study, eating speed and eating full were weakly correlated (Cremer’s coefficient association: 0.25), while other eating habits are not intercorrelated. Therefore, these unhealthy eating habits were independently associated with dyslipidemia, and the accumulation of these eating habits led to the higher prevalence of dyslipidemia.

The strength of this study is that it is the first to analyze the associations between self-reported eating habits and dyslipidemia using the data of a large number of young adult men and the first to comprehensively explore the impact of eating fast, eating after satiety, skipping breakfast, and eating out. An increasing trend of incidence coronary heart diseases was observed in a study comprising Japanese middle-aged men who worked for companies and lived in Osaka metropolitan areas [32], accompanied by an increased prevalence of overweight and elevated TC and abnormal glucose levels due to less physical activity and fat-rich diets [33], which are more prevalent in young people [34]. Therefore, the prevention and control of hyperlipidemia may contribute to the prevention of coronary heart disease.

However, our study has some limitations. First, the eating habits were self-reported and the questionnaire was not validated in the population we investigated. Regarding the eating speed, the self-reported eating speed data were fairly valid in Japanese: the percentage of exact and adjunct agreement was 46% and 47%, respectively [35]. Second, we collected non-fasting blood samples, but small differences between fasting and non-fasting samples for TC, HDL-C, and LDL-C [2, 15] could be present, but not for TG; thus, we used desirable concentration cut-off points for non-fasting lipids. Third, we did not collect the data on physical activity as a potential confounding factor. Less physical activity was associated with elevated TG and reduced HDL-C [2]. Instead, we adjusted for sedentary work as a surrogate marker of physical activity. Fourth, although we adjusted for many potential confounders, but there may be other unmeasured confounding variables. Lastly, a cross-sectional study does not assure causality, but our study suggested causality for two reasons. Many young adults do not modify their eating habits since dyslipidemia is generally asymptomatic, and they are less motivated to modify their eating habits than older adults. Moreover, little scientific evidence suggests that dyslipidemia causes changes in dietary habits.

## Conclusions

Eating fast, eating after satiety, skipping breakfast, and eating out were associated with a higher prevalence of dyslipidemia in men aged 20–39 years. Prospective cohort and intervention studies are needed to validate these associations between eating habits and the risk of dyslipidemia.

## Abbreviations

TC: total cholesterol
TG: triglyceride
HDL-C: high-density lipoprotein cholesterol
LDL-C: low-density lipoprotein cholesterol
OR: Odds ratio
CI: confidence interval
CVD: cardiovascular disease
JECS: Japan Environment and Children’s Study

## Declaration

### Availability of data and material

Data are unsuitable for public deposition due to ethical restrictions and legal framework of Japan. It is prohibited by the Act on the Protection of Personal Information (Act No. 57 of 30 May 2003, amendment on 9 September 2015) to publicly deposit the data containing personal information. Ethical Guidelines for Medical and Health Research Involving Human Subjects enforced by the Japan Ministry of Education, Culture, Sports, Science and Technology and the Ministry of Health, Labour and Welfare also restricts the open sharing of the epidemiologic data. All inquiries about access to data should be sent to: jecs-en@nies.go.jp. The person responsible for handling enquiries sent to this e-mail address is Dr Shoji F. Nakayama, JECS Programme Office, National Institute for Environmental Studies.

## References

[1] Roth GA, . Global Burden of Cardiovascular Diseases and Risk Factors, 1990–2019: Update From the GBD 2019 Study. J J Am Coll Cardiol. 2020;76(25):2982–3021. 33309175 10.1016/j.jacc.2020.11.010 PMC7755038

[2] Mach F, . 2019 ESC/EAS Guidelines for the management of dyslipidaemias: lipid modification to reduce cardiovascular risk. Eur Heart J. 2020;41(1):111–88. 31504418 10.1093/eurheartj/ehz455

[3] Yoshida S, . Development and validation of ischemic heart disease and stroke prognostic models using large-scale real-world data from Japan. Environ Health Prev Med. 2023;28:16. 36792224 10.1265/ehpm.22-00106 PMC9989775

[4] Iso H, . Fasting and non-fasting triglycerides and risk of ischemic cardiovascular disease in Japanese men and women: the Circulatory Risk in Communities Study (CIRCS). Atherosclerosis. 2014;237(1):361–8. 25443874 10.1016/j.atherosclerosis.2014.08.028

[5] Cui R, . Serum total cholesterol levels and risk of mortality from stroke and coronary heart disease in Japanese: the JACC study. Atherosclerosis. 2007;194(2):415–20. 16970954 10.1016/j.atherosclerosis.2006.08.022

[6] Andersson C, Vasan RS. Epidemiology of cardiovascular disease in young individuals. Nat Rev Cardiol. 2018;15(4):230–40. 29022571 10.1038/nrcardio.2017.154

[7] Pletcher MJ, . Nonoptimal lipids commonly present in young adults and coronary calcium later in life: the CARDIA (Coronary Artery Risk Development in Young Adults) study. Ann Intern Med. 2010;153(3):137–46. 20679558 10.1059/0003-4819-153-3-201008030-00004 PMC3468943

[8] Koetaka H, . Long-term effects of lifestyle on multiple risk factors in male workers. Environ Health Prev Med. 2009;14(3):165–72. 19568844 10.1007/s12199-008-0076-3 PMC2684801

[9] Maruyama K, . The joint impact on being overweight of self reported behaviours of eating quickly and eating until full: cross sectional survey. BMJ. 2008;337:a2002. 18940848 10.1136/bmj.a2002 PMC2572205

[10] Sakurai M, . Self-reported speed of eating and 7-year risk of type 2 diabetes mellitus in middle-aged Japanese men. Metabolism. 2012;61(11):1566–71. 22560127 10.1016/j.metabol.2012.04.005

[11] Kawamoto T, . Rationale and study design of the Japan environment and children’s study (JECS). BMC Public Health. 2014;14(1):25. 24410977 10.1186/1471-2458-14-25 PMC3893509

[12] Michikawa T, . Baseline Profile of Participants in the Japan Environment and Children’s Study (JECS). J Epidemiol. 2018;28(2):99–104. 29093304 10.2188/jea.JE20170018 PMC5792233

[13] Willett W. Nutritional epidemiology. Oxford University Press; 2012.

[14] Yokoyama Y, . Validity of Short and Long Self-Administered Food Frequency Questionnaires in Ranking Dietary Intake in Middle-Aged and Elderly Japanese in the Japan Public Health Center-Based Prospective Study for the Next Generation (JPHC-NEXT) Protocol Area. J Epidemiol. 2016;26(8):420–32. 27064130 10.2188/jea.JE20150064 PMC4967663

[15] Nordestgaard BG, . Fasting is not routinely required for determination of a lipid profile: clinical and laboratory implications including flagging at desirable concentration cut-points-a joint consensus statement from the European Atherosclerosis Society and European Federation of Clinical Chemistry and Laboratory Medicine. Eur Heart J. 2016;37(25):1944–58. 27122601 10.1093/eurheartj/ehw152 PMC4929379

[16] Lee K, . Eating rate is associated with cardiometabolic risk factors in Korean adults. Nutrition. 2013;23(7):635–41. 10.1016/j.numecd.2012.02.003 22633791

[17] Zhu B, . Association between eating speed and metabolic syndrome in a three-year population-based cohort study. J Epidemiol. 2015;25(4):332–6. 25787239 10.2188/jea.JE20140131 PMC4375288

[18] Tao L, . Association between self-reported eating speed and metabolic syndrome in a Beijing adult population: a cross-sectional study. BMC Public Health. 2018;18(1):855. 29996822 10.1186/s12889-018-5784-z PMC6042428

[19] Otsuka R, . Eating fast leads to insulin resistance: Findings in middle-aged Japanese men and women. Prev Med. 2008;46(2):154–9. 17822753 10.1016/j.ypmed.2007.07.031

[20] Kokkinos A, . Eating slowly increases the postprandial response of the anorexigenic gut hormones, peptide YY and glucagon-like peptide-1. J Clin Endocrinol Metab. 2010;95(1):333–7. 19875483 10.1210/jc.2009-1018

[21] Klonoff DC, . Exenatide effects on diabetes, obesity, cardiovascular risk factors and hepatic biomarkers in patients with type 2 diabetes treated for at least 3 years. Curr Med Res Opin. 2008;24(1):275–86. 18053320 10.1185/030079908x253870

[22] Terán-García M, . Effects of long-term overfeeding on plasma lipoprotein levels in identical twins. Atherosclerosis. 2004;173(2):277–83. 15064102 10.1016/j.atherosclerosis.2003.12.016

[23] Cornford AS, . Rapid development of systemic insulin resistance with overeating is not accompanied by robust changes in skeletal muscle glucose and lipid metabolism. Appl Physiol Nutr Metab. 2013;38(5):512–9. 23668758 10.1139/apnm-2012-0266 PMC3891585

[24] Johannsen DL, . Effect of 8 weeks of overfeeding on ectopic fat deposition and insulin sensitivity: testing the “adipose tissue expandability” hypothesis. Diabetes Care. 2014;37(10):2789–97. 25011943 10.2337/dc14-0761 PMC4170127

[25] Bray G, Bouchard C. The biology of human overfeeding: A systematic review. Obes Rev. 2020;21(9):e13040. 32515127 10.1111/obr.13040

[26] Bonnet JP, . Breakfast Skipping, Body Composition, and Cardiometabolic Risk: A Systematic Review and Meta-Analysis of Randomized Trials. Obesity. 2020;28(6):1098–109. 32304359 10.1002/oby.22791 PMC7304383

[27] Deshmukh-Taskar P, . The relationship of breakfast skipping and type of breakfast consumed with overweight/obesity, abdominal obesity, other cardiometabolic risk factors and the metabolic syndrome in young adults. The National Health and Nutrition Examination Survey (NHANES): 1999–2006. Public Health Nutr. 2013;16(11):2073–82. 23031568 10.1017/S1368980012004296 PMC10271246

[28] Harris IR, . Regulation of HMG-CoA synthase and HMG-CoA reductase by insulin and epidermal growth factor in HaCaT keratinocytes. J Invest Dermatol. 2000;114(1):83–7. 10620120 10.1046/j.1523-1747.2000.00822.x

[29] Russell DW. Cholesterol biosynthesis and metabolism. Cardiovasc Drugs Ther. 1992;6(2):103–10. 1390320 10.1007/BF00054556

[30] Lachat C, . Eating out of home and its association with dietary intake: a systematic review of the evidence. Obes Rev. 2012;13(4):329–46. 22106948 10.1111/j.1467-789X.2011.00953.x

[31] Toft U, . The Dietary Quality Score: validation and association with cardiovascular risk factors: the Inter99 study. Eur J Clin Nutr. 2007;61(2):270–8. 16929244 10.1038/sj.ejcn.1602503

[32] Kitamura A, . Trends in the Incidence of Coronary Heart Disease and Stroke and Their Risk Factors in Japan, 1964 to 2003: The Akita-Osaka Study. J Am Coll Cardiol. 2008;52(1):71–9. 18582638 10.1016/j.jacc.2008.02.075

[33] Iso H. Lifestyle and Cardiovascular Disease in Japan. J Atheroscler Thromb. 2011;18(2):83–8. 21307610 10.5551/jat.6866

[34] Ministry of Health, L. and Welfare. National Health and Nutrition Survey. 2020. Available from: https://www.mhlw.go.jp/bunya/kenkou/kenkou_eiyou_chousa.html.

[35] Sasaki S, . Self-reported rate of eating correlates with body mass index in 18-y-old Japanese women. Int J Obes Relat Metab Disord. 2003;27(11):1405–10. 14574353 10.1038/sj.ijo.0802425

